# A First Analysis of Metallome Biosignatures of Hyperthermophilic Archaea

**DOI:** 10.1155/2012/789278

**Published:** 2012-12-03

**Authors:** Vyllinniskii Cameron, Christopher H. House, Susan L. Brantley

**Affiliations:** ^1^Department of Geosciences and Penn State Astrobiology Research Center, The Pennsylvania State University, University Park, PA 16802, USA; ^2^School of Earth Sciences, University of Bristol, Bristol BS8 1RJ, UK

## Abstract

To date, no experimental data has been reported for the metallome of hyperthermophilic microorganisms although their metal requirements for growth are known to be unique. Here, experiments were conducted to determine (i) cellular trace metal concentrations of the hyperthermophilic Archaea *Methanococcus jannaschii* and *Pyrococcus furiosus*, and (ii) a first estimate of the metallome for these hyperthermophilic species via ICP-MS. The metal contents of these cells were compared to parallel experiments using the mesophilic bacterium *Escherichia coli* grown under aerobic and anaerobic conditions. Fe and Zn were typically the most abundant metals in cells. Metal concentrations for *E. coli* grown aerobically decreased in the order Fe > Zn > Cu > Mo > Ni > W > Co. In contrast, *M. jannaschii* and *P. furiosus* show almost the reverse pattern with elevated Ni, Co, and W concentrations. Of the three organisms, a biosignature is potentially demonstrated for the methanogen *M. jannaschii* that may, in part, be related to the metallome requirements of methanogenesis. The bioavailability of trace metals more than likely has varied through time. If hyperthermophiles are very ancient, then the trace metal patterns observed here may begin to provide some insights regarding Earth's earliest cells and in turn, early Earth chemistry.

## 1. Introduction

Trace metals are vital components of all living systems and are required for a host of metabolic and structural functions. Primary amongst these is a catalytic role in a vast array of cellular reactions including energy generation. A cell's elemental contents are defined to be its metallome: the major elements required for all life, plus approximately ten bioessential transition metals that include Fe, Mn, Co, Mo, Zn, Ni, Cr, Cu, V, and W. Some metals such as Fe and Zn have a universal utility in most organisms while others perform specific functions only in certain organisms [1].

Differences in metallomes may be broadly explained by diverse microbial requirements as well as the availability of elements that have varied in the environment through time (see, e.g., [2–4]). These variations, facilitated by changing environmental conditions on the Earth, most likely directly impacted the evolution of life. The evidence for this may be documented in variations in metal utilization amongst specific groups of organisms that evolved at different periods during Earth's history. For instance, Ni and W are fundamental requirements for the growth of anaerobic archaeal methanogens and hyperthermophiles, respectively, where they operate in metabolisms that are generally thought to be quite primitive [1, 5]. On the other hand, the use of Mo in bacteria and eukarya for nitrogen fixation and nitrate reduction was probably initiated after oxygenation of the Earth at ~2.2 Ga (e.g., [6, 7]). With the increase in oxygen in that atmosphere, Mo oxides with relatively high solubility became a more biologically available species [8, 9].

Hyperthermophiles grow optimally at temperatures >80°C and are found predominantly within the archaeal domain of life. There are two primary archaeal groups: the largely hyperthermophilic Crenarchaeota and the methanogen-containing Euryarchaeota. Hyperthermophiles often occupy the deepest and shortest lineages in both prokaryotic domains [10, 11] and are found in unique high temperature environments such as deep ocean hydrothermal vents or geothermal terrestrial sites [11, 12]. These natural environments typically contain relatively high, and in some cases, toxic concentrations of metals that paradoxically promote and sustain the growth of microorganisms. For example, the concentration of Fe^2+^ in seawater is generally <1 nM but can commonly exceed values of 10 mM in hydrothermal vent fluids [13–15]. However, all organisms including hyperthermophiles have specific strategies and evolved mechanisms for coping with environmental stressors. For instance, some microorganisms are capable of producing metal chelators which function to both secure metals for the cell or conversely, bind and remove harmful species intracellularly or extracellularly, for example, [16–22].

Our understanding of how concentrations of bioessential trace metals have changed in the environment over time has grown significantly. However, the same cannot be said for our understanding of the majority of archaeal prokaryotes or in fact, the extreme habitats in which they live. Likewise, the utilization of trace metals by various microbial groups is a function of many factors including availability, cellular requirements, environmental constraints, and substitution or competition between metals [23]. A large number of experimental studies have attempted to quantify the intracellular metal quotas of various, primarily marine, organisms. In all such investigations it is difficult to prove whether the reported metal quotas represent the true metallome of the organisms under study, or simply the media composition or specific growth conditions. Additionally, it is not known whether eukaryotic metal stoichiometries are also representative of prokaryotic quotas. For prokaryotes, these issues are exacerbated by the fact that microorganisms requiring different metals for different metabolisms also require different growth media. 

Examination of the correlations between trace metal utilization, microbial metabolisms, and the microorganisms themselves can be used to infer environmental changes through time and could possibly be exploited as potential biosignatures (e.g., [24–27]). The ability of early microorganisms to adapt and take advantage of varying metal conditions would have been beneficial in that it could have allowed the optimization of metabolic processes as well as the adaptation of microorganisms to new environments.

Towards these ends, a systematic census of the trace metal contents of the prokaryotic metallome needs to be undertaken to better understand the biogeochemical links between these organisms and the environments they inhabit. In this contribution, we investigated the trace metal contents of two hyperthermophilic microorganisms in order to (1) determine their trace metal contents; (2) investigate the cellular uptake of metals under variable metal concentrations; (3) explore the possibility of a metal biosignature for hyperthermophilic systems. As a baseline and in order to make comparisons and inferences, metal contents were also determined for *E. coli*.

## 2. Materials and Methods

### 2.1. Microorganisms

Three microorganisms were used in this study: the bacterium *E. coli*, a heterotrophic hyperthermophile *Pyrococcus furiosus*, and the hyperthermophilic methanogen *Methanococcus jannaschii*. The latter two are strict marine anaerobes; *E. coli* is a terrestrial facultative aerobe which was grown under aerobic and anaerobic conditions. The organisms were cultivated under their optimum growth temperatures: *E. coli*, 37°C; *P. furiosus*, 98°C; *M. jannaschii*, 85°C. All microorganisms were originally obtained from the Deutsche Sammlung von Mikroorganismen und Zellkulturen (DSMZ) and are maintained in the laboratory of C. H. House (Penn State University, University Park, PA, USA). *M. jannaschii* was recently reclassified to *Methanocaldococcus jannaschii* [28].

### 2.2. Growth Media

Given the extreme difficulty of growing hyperthermophiles as well as their complex nutritional requirements, it was not our intent to grow cells under complete metal limitation. However, attempts were made to culture the microorganisms as close to metal limitation as possible.

All labware used in media preparation and sample collection was cleaned by soaking in 10% HCl followed by thorough rinsing in 18 MΩ water. Reagents were supplied from Fisher or Sigma-Aldrich Chemicals. Savillex PFA labware (Minnetonka, MN, USA) was used to collect and digest all final cell pellets and cell lysate samples.

Organic substrates used in the *E. coli* and *P. furiosus* media were first treated with Chelex 100 resin (200–400 mesh, Biorad, CA, USA). This treatment removed metals contained in the organics and established a minimal metal baseline. Mineral stock solutions (see below) containing the various trace metals were made up separately and then added to the resin-cleaned organic solutions to make up the final growth media.

The *E. coli* medium contained (per liter) 10 g LB powder; 5x Na_2_WO_4_·2H_2_O mineral solution (0.02 g L^−1^); 5x mineral solution containing (per liter) 2.0 g MgSO_4_·7H_2_O, 0.2 g MnSO_4_·H_2_O, 0.2 g FeSO_4_·7H_2_O, 0.02 g CoSO_4_·7H_2_O, 0.1 g ZnSO_4_·7H_2_O, 0.02 g CuSO_4_·5H_2_O, 0.01 g Na_2_MoO_4_·2H_2_O, 0.02 g L^−1^ NiCl_2_·6H_2_O, 0.01 g L^−1^ Na_2_SeO_3_·5H_2_O, 0.01 g VOSO_4_, 0.01 g CrK(SO_4_)_2_·12H_2_O, 0.008 g H_3_BO_3_. Concentrated HCl was used to adjust the pH (~3) of the stock mineral solution to keep metals in solution. This medium was made under aerobic and anaerobic conditions. The LB powder was first dissolved in a volume of MQ water before Chelex 100 resin (~3.0 g L^−1^) was added; the mixture was stirred for 1 hr then gravity filtered to remove the resin. Trace metal solutions were then added and the medium was brought up to volume and adjusted to pH 7. Aerobic medium was dispensed into 1 L Erlenmeyer flasks that were capped with silicone plugs. Anaerobic medium was degassed by bubbling with a stream of N_2_ for ~20 min; pH adjustment with concentrated HCl or NaOH (10%) and dispensation into serum bottles was carried out in an anaerobic chamber. The headspace in the anaerobic bottles was flushed three times with N_2_, followed by a final addition of the same gas to 0.5 bar pressure. The growth of *E. coli* typically does not require additional trace metals in the growth medium but was included for some of these experiments (HM, Growth Experiments) to ensure uniformity between all trace metal experiments. The medium was sterilized by autoclaving.

The *P. furiosus* medium contained (per liter) 3.0 g Na_2_SO_4_, 0.2 g KH_2_PO_4_, 0.3 g NH_4_Cl, 0.3 g KCl, 0.2 g CaCl_2_·2H_2_O, 0.3 g MgCl_2_·6H_2_O, 20.0 g NaCl, 5.0 g peptone, 1.0 g yeast, and both 5x mineral solutions (see *E. coli* medium preparation). A solution of peptone, yeast, and Chelex 100 resin was stirred for 1 hr then gravity was filtered to remove the resin. The medium was then made by mixing all reagents into solution and degassed by bubbling with a stream of N_2_ for ~20 min. The pH (7) adjustment and dispensation into 0.5 L serum bottles was carried out in an anaerobic chamber. The headspace in the experiment bottles was flushed three times with N_2_, followed by a final addition of the same gas to 2 bars pressure. The medium was sterilized by autoclaving.

The *M. jannaschii* medium contained (per liter) 3.0 g Na_2_SO_4_, 0.2 g KH_2_PO_4_, 0.3 g NH_4_Cl, 0.3 g KCl, 0.2 g CaCl_2_·2H_2_O, 0.3 g MgCl_2_·6H_2_O, 20.0 g NaCl, 3.0 mL NaOH solution (10%) and both 5x mineral solutions (see *E. coli* medium preparation), 0.5 g cysteine·HCl. Medium was made by mixing in solution all reagents except cysteine and degassed by bubbling with a stream of H_2_ + CO_2_ (80% + 20%) for ~20 min. The addition of cysteine, pH adjustment (7), and dispensation into 1 L serum bottles was carried out in an anaerobic chamber. The headspace in the experiment bottles was flushed three times with H_2_ + CO_2_, followed by a final addition of the same gas mix to 2 bars pressure. The medium was sterilized by autoclaving.

### 2.3. Growth Experiments

In order to estimate the metallome of the microorganisms in this study, two approaches were taken to extract cellular trace metals: whole cell acid digestion and a suite of cell lysis techniques. Details are provided below for all techniques.

Cells were grown under three separate trace metal concentrations: no metal (NM), low metal (LM), and high metal (HM). The latter two formed the basis of lysis experiments but were not the primary focus here (see Supplementary Material available online at doi:10.1155/2012/789278). Whole cell acid digestion of all microorganisms was only carried out for the NM experiments while lysis experiments were conducted for all three metal conditions but not for every cell. NM means that trace metals were not added to the growth media for these experiments. However, *M. jannaschii* will not grow in a medium without trace metals. Therefore, the NM medium for *M. jannaschii* was made with the same concentration of added trace metals as the LM batch but was filtered (0.1 *μ*m Acrodisc syringe filter) after autoclaving. Pure culture stocks of all microorganisms were grown in the base media without the addition of trace metals (NM). These cultures were established prior to being used as inoculum for the growth experiments. Media were sampled prior to inoculation at the beginning of growth. Depending on the microorganism and trace metal condition, growth of various cells typically proceeded over a period of 2 to 4 days. *P. furiosus* and *E. coli* had the fastest growth times (maximum 2 days) and *M. jannaschii*, the longest. *P. furiosus* and *E. coli* usually have doubling times on the order of minutes under optimum growth conditions and the increased growth period observed in these experiments may be due to a longer lag phase as the cells acclimatize to the growth media.

Following growth, the entire volume of cells and spent medium (supernatant) were gently mixed and poured into centrifuge tubes. Samples for cell counts and supernatant analysis were removed before centrifuging (5000 rpm, 1 hr, 10°C). Another supernatant sample was removed after centrifuging, the medium was decanted, and the cell pellet washed 3 times with an ultrapure NaCl solution. The salt solution used for washing was analogous to the salt (NaCl) concentration in the medium for each cell (*M. jannaschii* and *P. furiosus*, 2.5%; *E. coli*, 0.5%). During the wash steps, cells were centrifuged at 8000 rpm for 15 min at 10°C. After washing, the bulk cell pellet was slurried in ultrapure water and divided into equal volume fractions for acid digestion and lysis. Cell numbers were determined via direct cell counting with a light microscope.

The cell pellet fraction for acid digestion was centrifuged to remove most of the liquid and the pellet transferred into PFA beakers and dried (50°C) to a constant weight before digestion (see below). Three cell lysis techniques were employed: ultrasonication, freeze-thaw, and bead beating. The conditions for each technique are as follows. A Branson Sonifier 450 (output ~3) was used for ultrasonication. The tip was cleaned with ethanol and 18 MΩ water before being used. Samples were sonicated for 15 sec followed by a cooling period of 45 sec in an ice-water slurry; this process was repeated 3 times. For the freeze-thaw method, the cell pellet slurry was first frozen at −80°C overnight, removed the next day, and allowed to thaw at room temperature before being gently mixed and frozen again; this was repeated 3 times. Zirconium oxide beads (low-binding, 200 *μ*m, OPS Diagnostics LLC, NJ) were used in the bead beating technique. The beads were cleaned in 50% ultrapure HNO_3_, rinsed with double distilled water, and dried prior to being used. The cell pellet was homogenized for 5 min then cooled for 3 min in an ice bath; this was repeated over a period of 20 min. The lysed cells from the above three methods were centrifuged (5000 rpm, 30 min) and the lysates filtered through 0.1 *μ*m Acrodisc syringe filters directly into PFA beakers. Lysis experiments were not conducted for *M. jannaschii*—NM (entire volume of cells acid digested) and *E. coli*—LM fractions.

Media, supernatant, and experimental blank samples were filtered (0.1 *μ*m Acrodisc filters) and acidified with 2.5%  HNO_3_ + 0.1% HF for analysis. The cell lysates and dried cell pellets were digested overnight in concentrated ultrapure HNO_3_ and finally taken up in 2.5%  HNO_3_ + 0.1% HF. Media and supernatants were typically diluted 50x for analysis while the cell pellets and lysates were analyzed undiluted. Indium (In) was used as an internal standard to monitor analyte sensitivity and was added to all samples and standards to a concentration of 20 ppb before analysis. External standardization was carried out by constructing standard calibration curves to determine detection limits and linear working ranges of major and trace elements; this process involved making up three separate sets of matrix-matched standards (2.5, 0.5 & 0% NaCl) and pure standard solutions over a range of concentrations utilizing individual cation stocks (High-Purity Standards) and a multielement trace element stock (PSU-CAL-1, Inorganic Ventures, Inc.). Standards were analyzed prior to each analytical session. Element concentrations were measured on a high resolution inductively coupled plasma mass spectrometer (HR-ICP-MS, Finnigan Element 1). Estimated uncertainties in concentration values are 10%. Detection limits (*μ*g/L), calculated as 3x the SD of the blanks for all experiments for each microorganism, were in the following ranges for trace metals reported: Fe (1.0–5.0), Zn (0.1–5.0), Mn (0.01–0.5), Ni (0.3–1.0), Mo (0.1), Cu (0.01–0.05), W (0.01), Co (0.01–0.1).

## 3. Results

Given the criteria of growth under low metal concentrations, the NM experiments were the primary focus in this study, particularly the whole cell acid digested fractions. The following results and discussion are therefore centered on the NM data, with additional results regarding most of the lysis experiments (LM and HM) provided in the Supplementary Material.

Metal concentrations measured in the NM growth media are shown in Table 1. Residual amounts of metals, particularly Fe and Zn, are probably more abundant in the organic substrates used for the *E. coli* and *P. furiosus* media and were possibly not as effectively removed after treatment with Chelex. Nevertheless, excluding Fe and Zn, the background trace metal content of all NM media is relatively low (<0.1 mg/L). Note that, as mentioned previously, the NM *M. jannaschii* medium was not treated with Chelex but metal concentrations at the LM level were added and the medium was filtered to remove metal colloids and/or inorganic precipitates, which would reduce the overall concentration of metals. In all of the experiments most trace metals were effectively washed off the cells by wash step 2 (Table S2). The total growth volumes and resulting cell numbers are presented in Table 2. The largest cell pellet (i.e., with the greatest number of cells) was obtained for the aerobic *E. coli* cultures. The smallest was attained for the hyperthermophiles *P. furiosus* and *M. jannaschii*.

The growth patterns observed are not unexpected especially in the case of *E. coli* which does not typically require additional trace metals for cell growth but instead obtains all bioessential nutrients from the organic medium in which it is grown. By comparison, most hyperthermophiles are commonly grown in the laboratory in an inorganic medium or an organic-supplemented inorganic medium containing sufficient levels of added trace metals required for optimum growth. Additionally, microorganisms are typically cultured under prescribed optimum conditions but limited growth may occur regardless of cell acclimation to nonoptimal conditions. This may have been the case for the facultative microbe *E. coli*, where higher cell numbers were obtained for the cells grown aerobically (this cell's preferred mode of growth) compared to the anaerobic cells.

**Figure 1:**
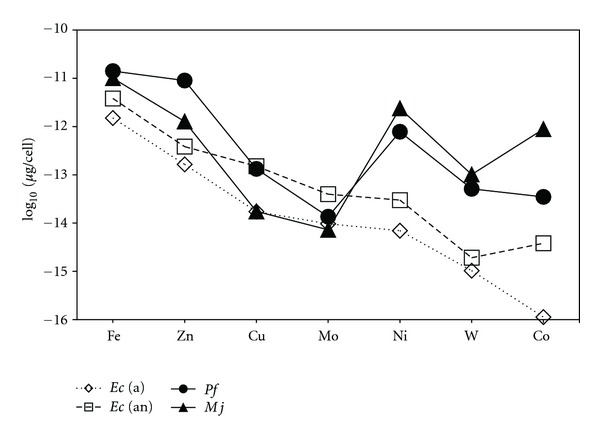
Calculated metal concentrations [*μ*g/cell] for all microorganisms from the *whole cell acid digest* fractions of the NM experiments. Trace metals are placed in order of decreasing concentration measured for *E. coli *(a). Metal concentrations for the other cells are plotted relative to the order of the *E. coli *(a) values. Metal abundance per cell was calculated using the analyzed whole cell concentrations and cell data reported in Table 2. *E. coli* aerobic *Ec* (a); *E. coli* anaerobic *Ec* (an); *P. furiosus Pf*; methanogen, *M. jannaschii Mj*.

Whole cell metal concentrations are interpreted as the metallome of the cells. These metallomes are summarized for the acid digest fractions of the NM experiments in Figure 1. Fe was the most abundant metal in all cells however, there is a marked contrast in the other metal contents between cells. Of particular interest is the metal content of the methanogen *M. jannaschii*. Excluding Mn which was below detection for the acid digest fractions, the order of metal abundance (*μ*g/cell) for *M. jannaschii* is Fe > Ni > Co > Zn > W > Cu > Mo while that of *E. coli* is almost the reverse; Fe > Zn > Cu > Mo > Ni > W > Co. The metal contents measured for *P. furiosus* lie between the trends observed for the other two microorganisms: Fe > Zn > Ni > Cu > W > Co > Mo. The trend observed for *E. coli* grown under the presence or absence of oxygen was essentially the same; the only variations noted are slightly higher metal concentrations, particularly for Co, in the anaerobically grown cells. In general, the concentrations of most metals in the hyperthermophiles are to some degree enhanced over *E. coli*. However, hyperthermophilic concentrations of Ni, Co, and W were conspicuously greater, by at least an order of magnitude, compared to the bacterium.

**Figure 2:**
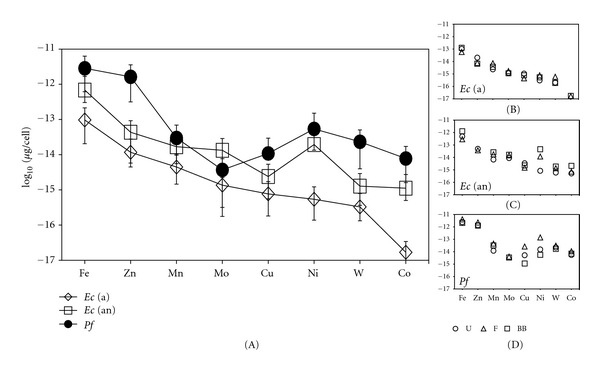
Results from the NM (no metal) lysis experiments. Metals are placed in order of decreasing concentrations measured for aerobic *E. coli *(a). Metal concentrations for *E. coli* anaerobic *Ec *(an) and *P. furiosus Pf* are plotted relative to the order of the *E. coli *(a) values. Panels (B), (C), and (D) show the metal concentrations measured for each lysis technique, for each microorganism. The values from all three lysis methods were then averaged and used to calculate ±1*σ* error bars, which are shown in panel A. Symbols for the lysis methods (below panel (D)) are the same for all cells: U, ultrasonication; F, freeze-thaw; BB, bead beating.

Metal concentrations measured for the three physical lysis techniques (ultrasonication, freeze-thaw, bead beating) of the NM experiments for all cells show small and generally insignificant variations (Figure 2). In general, these data mirror the whole cell data in Figure 1. Only Cu and/or Ni showed evidence for differences between the lysis techniques, and this was observed for *E. coli* (anaerobic) and *P. furiosus*. Furthermore, a few differences are noted in the hierarchy of metals for the lysis techniques when compared to the acid digest results but the trends are similar. Variations are most likely attributable to small differences imparted by the individual lysis methods. There is some variation in concentration between the NM whole cell and lysis fractions (Figures 1 and 2). Metal concentrations are generally higher in the former which could be due to a greater degree of metal release achieved by the acid digest method and/or losses that could have occurred during filtration of the lysates. Metals found in cellular membrane fractions removed during filtration could also account for losses, in particular Cu and Zn cytoplasmic concentrations have been shown to be tightly controlled, with the bulk of these metals complexed with various storage proteins until needed [29–31].

## 4. Discussion

Disregarding Fe and Mn, the trace metal trends for the *E. coli* aerobic and anaerobic NM experiments are almost identical. Concentrations of metals in all experiments for this bacterium were in the range 1 × 10^−11^ to 1 × 10^−17^ *μ*g/cell (Figures 1 and 2). Based on these observations, the metals in order of abundance in *E. coli* regardless of aerobic or anaerobic growth are Fe > Zn > Mn, Cu > Mo > Ni > W > Co. A similar trace metal pattern for measured metals, for the *E. coli* metallome has been previously reported by Outten and O'Halloran [30]. In that study, minimal and rich media were utilized which are approximated by the NM and HM experiments, respectively, in this study. The HM lysis data is shown in Figure S2. In general, the metal quotas reported in both studies are quite comparable with minor variations possibly being attributable to differences in culturing techniques or the individual lysis/experimental methods.

Trace metal concentrations for the hyperthermophile experiments were in the range 1 × 10^−11^ to 1 × 10^−15^ *μ*g/cell. *M. jannaschii* showed a clear hierarchy of metal concentrations that decreased in the order Fe > Ni > Zn, Co > W, Cu > Mo (Figure 1). Once again, *P. furiosus* had metal patterns in common with both of the other microorganisms though these were more distinct than of *E. coli*. The metals in order of abundance to *P. furiosus* are Fe > Zn > Ni, W > Cu, Co > Mo (Figures 1 and 2).

An increase in the concentration of Ni was observed for *P. furiosus* in the NM experiments. Nickel is a required element in NiFe hydrogenases, one of the three major classes of enzymes catalyzing the reversible oxidation of H_2_. Hydrogenases are found in most prokaryotes and in diverse metabolisms. Contrary to the catalytic activity carried out by the majority of hydrogenases, the three NiFe hydrogenases found in *P. furiosus* all function in the generation of H_2_ when elemental sulfur is absent. These enzymes are not biosynthesized in the presence of S^0^ [32]. Elemental sulfur is typically used for the growth of *P. furiosus* which results in the cell reducing S^0^ to H_2_S but S^0^ was not used for the experiments conducted in this work. As such, the relative increase in Ni concentration observed for the growth of this cell in the metal limited experiment may be due to increased scavenging and uptake of Ni to meet metal quotas required for the expression of these enzymes.

An array of studies have documented the impact, utility, and interactions between trace metals and biological processes in modern environments including the biogeochemical cycling of metals (e.g., [16, 33–37]), weathering processes (e.g., [38–42]), mineral transformations (e.g., [43–47]), microbial metabolisms (e.g., [48–54]), and biomarkers (e.g., [55–59]). Such works have also led to a greater understanding of life on the early Earth, prevailing environmental conditions, and the evolution of these systems through time (e.g., [3, 7, 60–65]). More commonly, microbial trace metal investigations usually target specific cellular characteristics like enzymes [5, 66], metabolic functions [53, 67] or other processes, such as optimizing growth yields or determining metal concentrations under variable environmental factors (e.g., [17, 30, 52, 68]). In addition to focusing on just one or two specific metals, many of these studies typically involve individual species or type microorganisms like *E. coli* for which there might already be a library of relevant information.

Metal quotas documented in marine organisms have many effects including influencing growth rates and impacting both major and minor biogeochemical cycles in the natural environment. For example, laboratory investigations of trace metal concentrations, particularly in eukaryotic marine organisms, have shown that in general, the average metal stoichiometry represented by the extended Redfield ratio is proportionally regulated in species from different taxa [23, 69]. Yet, it is not entirely clear whether such data represents the physiological (biochemical) needs of the organisms as opposed to the composition of the growth media, culturing conditions, or differences between organisms. One successful strategy that has been used to disentangle metallome requirements from dictates related to media composition is to grow organisms in experiments designed to explore growth at the lower limits of metal concentrations (e.g., [70, 71]), a strategy particularly useful for organisms that are relatively easy to grow in the laboratory. Furthermore, an examination of the variations in metal uptake across disparate groups of organisms can be accomplished by growth experiments utilizing the same starting suite and concentration of metals. Such an approach was utilized in this study.

The trace metal data produced in the present study contributes valuable information regarding microbial metallomes, particularly for archaea. The vast majority of metallomics literature is based on experiments involving eukaryotic marine organisms such as phytoplankton and diatoms (e.g., [69, 71–74]). These studies have been critical to understanding the myriad of biogeochemical processes in the ocean, from controls on primary productivity to biological controls on ocean chemistry. One of the most important means by which metal quotas are determined is via measurement of the unchelated metal concentration over a range of concentrations. Growth media for marine organisms typically use chelating agents like EDTA to buffer metal concentrations; this mimics the organism's natural growth media (seawater) but also facilitates maximal growth rates [23]. EDTA was not used in this study (nor indicated in [30]). Certainly, some proportion of metals in our experiments were chelated by organic ligands in the organic-rich media of *E. coli* and *P. furiosus* but most metals should be present as labile bioavailable complexes. Cysteine, which can also chelate metals, was used in the inorganic medium of *M. jannaschii* but its function here is primarily as a required reducing agent and as a reduced source of sulfur. It is also a better substrate than inorganic Na_2_S as it causes less metal precipitation and in some studies, has been shown to increase the bioavailability of metals by increasing the formation of dissolved metal complexes [75]. 

**Figure 3:**
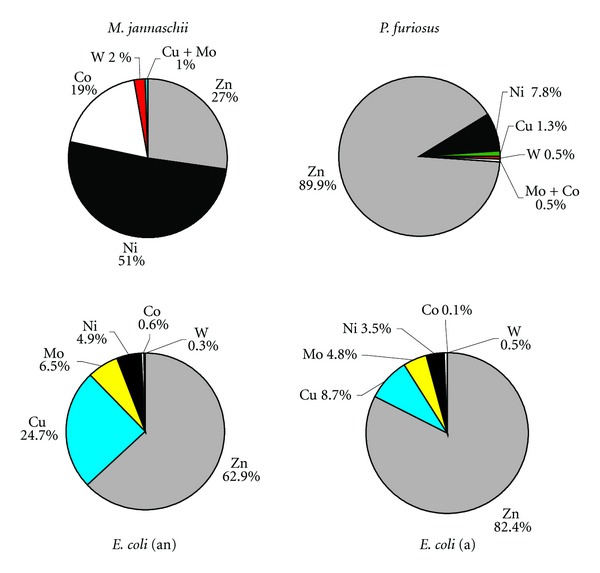
Pie charts depicting relative microbial abundances of reported metals other than Fe. Iron, as the most abundant metal in all cells, has not been included here to show the apparent differences in the cellular abundances of minor trace metals. The percentages are based on the *whole cell acid digest fraction* of the NM experiments. The trace metal pattern for the methanogen *M. jannaschii* is particularly distinct when compared to the other two microorganisms and may be useful as a potential biosignature.

Our data shows metal quotas are in general regulated over the range of experimental trace metal concentrations and as such, are at least partly determined by cellular requirements and not dictated entirely by the growth media. This observation is reinforced by comparing the gross elemental patterns for all microorganisms in the NM and HM (Figure S2) experiments. The overall abundance data for *E. coli* and *P. furiosus* produced in our study (Figure 3) also match well to the relative abundance amounts determined from cytoplasmic metalloproteins in Cvetkovic et al. [76] for the same microorganisms. Similar conclusions can be made for *M. jannaschii* when compared to the trace elemental compositions first reported by Scherer et al. [77].

The cellular trace metal concentrations are undoubtedly affected by a number of factors that can all influence a cell's metallome. Many of these factors come into play during growth and under specific growth or environmental conditions. For instance, numerous studies report the benefits of adding appropriate metal nutrients to stimulate growth and increase biomass (e.g., [52, 53]), while others describe methods that increase metal bioavailability (e.g., [75]). Unlike the vast literature for eukaryotes, luxury uptake studies in microbial systems are rare. The few studies that exist are focused on the uptake of macronutrients (C, N, P) rather than metal micronutrients; for instance, luxury uptake of phosphorus has been reported for sediment bacteria [78] and a methanogen [79]. Additional factors that can affect the metallome include uptake or inhibition of various metals which can substitute or compete with other metals in cellular functions. However, the most important criterion affecting metal uptake may be due to biochemical need [23]. Ample studies have shown the requirement for Ni and Co by methanogens (e.g., [67, 80]), while Cvetkovic et al. [76] found a specificity for Ni by a novel Ni-protein in *P. furiosus*, which when expressed in *E. coli* utilized Ni, Zn, or Co when this cell was grown in medium supplemented with the most abundant amounts of one of these metals, respectively. In this study, an obvious requirement for Ni and Co by *M. jannaschii* has been observed as well as a need for Ni by *P. furiosus*. Metabolic differences (e.g., heterotrophs/autotrophs, anaerobes/aerobes, mesophiles/hyperthermophiles) between microorganisms from the same species can also display variations in metal preferences. For instance, Sowers and Ferry [80] report Mo did not stimulate growth of *M. methylutens* but was essential for the growth of two other methanogens. *E. coli* in our study had increases in concentration of Cu, Mo, Ni, and Co (Figures 1 and 3) when grown under anaerobic but not aerobic conditions. In general, we believe the data presented here for most metals represents the individual metallomes of the microorganisms studied. The cellular values of metals measured in diverse groups of these hyperthermophilic taxa may vary by one or two orders of magnitude as has been shown for metal quotas for other organisms [69] and as such, the concentrations reported in this study may represent approximate average quotas for these microorganisms.

 Previous studies [2, 81, 82] have suggested that the cellular free metal ion concentration of bioessential elements are controlled and effectively maintained in the cytoplasm at very low levels, at values analogous to concentrations in the ocean. Additionally, these concentrations have been in place since the metallome system in primitive cells evolved with cells responding to the uptake and utilization of different and specific trace metals as (1) microorganisms evolved and (2) in response to the changing conditions of metal availability and concentrations. For instance, a constant Fe^2+^ concentration of 10^−7^ M is required by modern organisms [81] and is believed to have been required throughout life's evolution even when conditions on the Earth changed and the atmosphere became oxygenated. This concentration would have been established early in life's evolution as a result of factors such as the abundant Fe concentration present in a predominantly reducing environment as well as the binding constants for the metal established internally within the cell and externally in the ocean (e.g., [2, 62, 81]). Other elements such as Zn and Cu previously sequestered in the preoxidized, sulfidic ocean as inorganic sulfide precipitates became available after oxygenation at much higher toxic concentrations and early microorganisms adapted by constraining the cytoplasmic levels of these metals, <10^−10^ M and 10^−15^ M for Zn and Cu, respectively [81]. Relative to the seawater concentrations of redox-sensitive Fe, Cu, and Mo, the concentration of Ni, Co, and W may not have changed much through Earth's early transforming environment [1]. More importantly, these latter metals probably remained at bioavailable levels regardless of factors like changes in source inputs to the ocean or sequestration due to changing redox conditions.

The changes occurring in Earth's early environment from reducing to oxidizing states also allowed for microbial adaptations and utilization of previously unavailable metals, either in the capacity of coping with the changing environment and/or to optimizing metabolic functions previously met by other trace metals which could have facilitated the organism's evolution and radiation into new habitats. Alternatively, certain early microorganisms may have been restricted to habitable sites specifically because of shifting redox conditions and not necessarily because of a shortage of bioessential elements. Molybdenum and tungsten are two metals that particularly exemplify these ideas. Hot environments like deep ocean hydrothermal vents are natural habitats for hyperthermophilic microorganisms. These environments, thought to be a possible “cradle of life” [83–85] are characterized by reducing conditions and abundant trace metals such as W [13, 86, 87]. The behavior of Mo and W in the environment is dissimilar due to the redox chemistry of both metals [88]. Due to these properties, as well as its known utilization in anaerobic carbon metabolism and probably, high temperature enzyme stability [18, 89, 90], it has been postulated (e.g., [5]) that W may have been biologically available and thus had greater utility for primitive prokaryotes on the preoxygenated Earth. In contrast, Mo is expected to have been unavailable in the early oceans and would only have achieved its modern-day abundance and biological status after the onset of oxic conditions.

In modern environments, Mo is a key metal required for nitrogen, sulfur, and carbon metabolism, amongst others. For example, the Mo-containing nitrogenase is the most important of the three enzymes known to carry out nitrogen fixation; the other two (containing V and Fe) are less-efficient, not expressed when Mo is abundant and, are not used exclusively by any known organism [8]. Additionally, several molybdo-and tungsto enzymes and isoenzymes exist; however, “true” tungstoenzymes which can only utilize W have only been found in hyperthermophilic Archaea. Interestingly, analogous Mo enzymes are found in microorganisms like *E. coli* however, W substitution renders the enzymes inactive [5]. In line with all of these studies, the results of this work show W to be much more important than Mo to the hyperthermophiles, while the reverse is true for *E. coli*. Even though the dataset is small, a perceptible hierarchy of metals is demonstrated for each microorganism. The order of abundance of trace metals indicated by their relative concentrations and hence, utility in the cells, could be used to interpret and provide support for the evolution of these microorganisms and their metabolisms.

Of the three microorganisms investigated, the most promising biosignature may be the metallome trend observed for the hyperthermophilic methanogen, *M. jannaschii*. As shown in Figure 3, the high percentage of Ni, Co, and possibly W is distinctive for this cell when compared to the metallome percentages and trends of the other cells. Similar data based on a model metallome were previously reported [27]. Nickel is required by all methanogens specifically for carrying out methanogenesis. To date, at least three of the nine known Ni-dependent enzymes are found in methanogens and two are absolutely critical. The Ni-containing cofactor, F_430_ of MCR (methyl-coenzyme M reductase) catalyzes the terminal step in methanogenesis, the reduction of a methyl group to methane while the CODH/ACS (carbon monoxide dehydrogenase/acetyl coenzyme A synthase) catalyzes the oxidation of CO to CO_2_ as well as the formation or degradation of acetyl-CoA [91]. The cofactor F_430_ is unique to methanogens and has not been found in any other organism. Interestingly, hyperthermophilic methanogens also contain another exclusive enzyme, the W-containing FMDH (formylmethanofuran dehydrogenase) which catalyzes the initial conversion of CO_2_ at the beginning of methanogenesis. Mo-containing FMDHs are also found in some mesophilic to thermophilic methanogens [5].

Cobalt is another trace metal that has importance in metabolisms that have a probable primitive origin. The metal is found in the vitamin B12 cobalamin cofactor, in the same enzyme family as the Ni-containing F_430_ and is utilized primarily in anaerobic systems for metabolizing H_2_, CO, and –CH_3_ groups [1]. Not surprising then that Co displays such an enhanced concentration in *M. jannaschii*. There are two complex pathways for the *de novo* synthesis of cobalamin: the anaerobic route which does not require oxygen and the aerobic route in which molecular oxygen is required. These systems are only utilized by a few groups of prokaryotes and are not known to be present in eukaryotes [92, 93]. In fact even microorganisms like *E. coli* only induce the synthesis of cobalamin as well as Ni uptake under anaerobic conditions [1, 94], evidence for which is clearly demonstrated in this study.

## 5. Conclusion

The trace metal abundances of three distinctive microorganisms were determined, with the aim of characterizing the metallome content and hence, metal utilization of the cells. Metal concentrations were analyzed from whole cell digests and cellular lysates evolved from three physical cell lysis techniques applied to hyperthermophilic Archaea and the mesophilic bacterium, *E. coli*, which was grown under aerobic and anaerobic conditions. The results show metallome patterns for each microorganism but a distinctive pattern is particularly evident for the hyperthermophilic methanogen, *M. jannaschii* (Fe > Ni > Zn, Co > W, Mo > Cu). A potential biosignature is also suggested for this microorganism based on its unique metabolism that most likely relies to a great extent on the presence and availability of the trace metals Ni, Co and W. In contrast, the pattern of metal utilization observed for *E. coli* (Fe > Zn > Mn, Cu > Mo > Ni > W > Co) is more variable, possibly because of differing enzyme expression under different growth conditions. The metallome of the heterotrophic hyperthermophile *P. furiosus* (Fe > Zn > Ni, W > Cu, Co > Mo) is closer to that of *M. jannaschii* but displays some similarities in metal usage with *E. coli*. While not absolutely rigorous, the data produced in this study provides an estimated trace metal baseline for assessing future metallome studies involving archaeal and bacterial groups. 

The bioavailability of trace metals more than likely has varied through time yet the microbial metallome observed in modern extant cells may have been set at the time and in the environment, of the primitive ocean. Evolving microorganisms may have taken advantage of changing metal conditions by adapting and incorporating more available metals especially for improved metabolic and/or cellular functions or as a means of survival which could have facilitated their expansion into new environments and habitats. Conversely, changing redox conditions on the Earth may also have directly impacted early life regardless of any changes to the bioavailability of required elements. Therefore, microbial utilization of certain trace metals may provide information regarding the antiquity and evolution of microbial groups as well as their metabolisms.

## Supplementary Material

Data are presented for the physical lysis experiments conducted for the low metal (LM) and high metal (HM) experiments. The LM experiments involved only *P. furiosus* and *M. jannaschii* (Tables S1, S2, S3; Figure S1). In addition to these hyperthermophiles, the HM experiments also included *E. coli* grown aerobically and anaerobically (Tables S1, S2, S3; Figure S2). Details of all methods are described in the manuscript text.

## Figures and Tables

**Table 1:** Metal concentrations measured in the NM (no metal) growth medium for each microorganism. The values are the initial or starting concentrations in each medium, prior to cell inoculation. Concentrations were measured via ICP-MS. *E. coli* aerobic *Ec* (a); *E. coli* grown under anaerobic conditions *Ec* (an); *P. furiosus*; methanogen, *M. jannaschii*. Some values are below detection (BD): *M. jannaschii* detection limits for Zn and Cu are 5 and 0.05 *μ*g/L, respectively.

Microbial growth medium	Metal concentration (*μ*g/L)							
	Zn	Fe	Mn	Cu	Mo	Ni	Co	W
*E. coli *(a)	488.13	260.08	7.59	6.18	4.95	3.71	3.56	0.93
*E. coli *(an)	404.00	258.18	7.66	0.53	3.99	4.30	3.35	0.88
*P. furiosus *	352.55	24.63	1.84	0.19	10.14	3.10	2.27	0.28
*M. jannaschii *	BD	614.60	1229.63	BD	71.76	94.74	79.27	0.90

**Table 2:** Media volumes and cell pellet data for the NM experiment.

Microorganism	Media volume (mL)	Cell density (cells/mL)	Dry cell pellet weight (g)	Number of cell fractions
*E. coli *(a)	1000	1.09 × 10^11^	0.4392	4
*E. coli *(an)	1000	7.52 × 10^9^	0.0792	4
*P. furiosus *	1000	2.31 × 10^9^	0.0296	4
*M. jannaschii *	500	3.92 × 10^9^	0.0428	1

Cell densities were determined via direct cell counting. Calculation of cell numbers for individual fractions involves the total medium volume and cell density reported above, as well as the number of fractions that the respective pellet was divided into. The dry cell pellet weight was only determined for the acid digest cell fraction. *E. coli* aerobic *Ec* (a); *E. coli* anaerobic *Ec* (an); *P. furiosus*; *M. jannaschii*.
