# Supplementary material for: A First Analysis of Metallome Biosignatures of Hyperthermophilic Archaea
Source: Archaea. 2012 Dec 3;2012:789278. doi: 10.1155/2012/789278 (PMC3518089; doi:10.1155/2012/789278)
Supplement: Supplementary file 1 — Data are presented for the physical lysis experiments conducted for the low metal (LM) and high metal (HM) experiments. The LM experiments involved only P. furiosus and M. jannaschii (Tables S1, S2, S3; Figure S1). In addition to these hyperthermophiles, the HM experiments also included E. coli grown aerobically and anaerobically (Tables S1, S2, S3; Figure S2). Details of all methods are described in the manuscript text. [file 789278.f1.pdf]

Supplemental Data

Figures and Tables

Table S1: Metal concentrations [mg/L] in growth media utilized for the low (LM) and relatively higher (HM) trace metal experiments. The LM lysis experiments were only carried out for the hyperthermophiles, *P. furiosus* and *M. jannaschii*. Concentrations were measured via ICP-MS. Values for Cu for M. jannaschii are below detection (BD) at a detection limit of 0.05 μg/L.

LM.

Metal	*P. furiosus*	*M. jannaschii*
**Mn**	6.33E-01	6.06E-01
**Fe**	4.03E-01	3.81E-01
**Zn**	3.95E-01	2.76E-01
**Mo**	4.35E-02	4.25E-02
**Ni**	7.13E-02	5.94E-02
**Cu**	5.14E-02	BD
**W**	7.66E-02	1.10E-01
**Co**	4.43E-02	4.17E-02

HM.

Metal	*E.coli* (a)	*E.coli* (an)	*P. furiosus*	*M. jannaschii*
**Mn**	2.23E+00	1.90E+00	2.04E+00	1.93E+00
**Fe**	3.06E+00	2.61E+00	1.30E+00	1.14E+00
**Zn**	8.01E-01	3.31E-01	1.22E+00	1.12E-01
**Mo**	1.47E-01	1.73E-01	1.86E-01	1.61E-01
**Ni**	1.85E-01	1.63E-01	2.02E-01	1.87E-01
**Cu**	1.14E-01	1.22E-03	1.68E-01	BD
**W**	1.79E-01	2.06E-01	2.38E-01	2.05E-01
**Co**	1.66E-01	1.52E-01	1.50E-01	1.30E-01

Table S2: Metal concentrations [mg/L] in wash solutions (NaCl) after cells were washed. Concentrations were measured in samples of the wash solutions which were taken after cells were washed, centrifuged and pelleted (see text methods). For most experiments, data are for the solutions after wash step 2, but for **M. jannaschii* LM and HM, the results shown are from wash solutions taken after the third and final wash. Concentrations were measured by ICP-MS. A number of values were below detection (BD), for which the following detection limits (µg/L) are as follows: Mn [0.01-0.5]; Fe [1.0-5.0]; Zn [0.1-5.0]; Ni [0.3-1.0]; Cu [0.01-0.05]; W [0.01].

Metal	NM				LM		HM			
	E.coli (a)	*E.coli* (an)	*P. furiosus*	*M. jannaschii*	*P. furiosus*	**M. jannaschii*	*E.coli* (a)	*E.coli* (an)	*P. furiosus*	**M. jannaschii*
**Mn**	3.70E-04	1.53E-03	BD	2.55E-03	4.65E-03	6.20E-04	4.02E-01	2.68E-01	2.83E-02	3.38E-02
**Fe**	3.05E-03	1.81E-02	BD	BD	BD	BD	2.80E-02	3.73E-02	BD	BD
**Zn**	BD	BD	BD	5.46E-03	6.07E-02	6.34E-03	1.13E-02	6.58E-03	1.42E-01	BD
**Mo**	1.06E-03	8.03E-05	5.23E-05	8.44E-05	9.05E-04	7.50E-05	7.55E-03	2.25E-03	3.60E-03	7.49E-03
**Ni**	1.72E-04	BD	BD	BD	BD	BD	2.91E-03	1.05E-03	3.97E-04	1.23E-03
**Cu**	5.92E-04	1.18E-03	BD	BD	5.34E-03	BD	2.66E-03	4.82E-04	1.36E-02	BD
**W**	BD	BD	1.70E-05	2.19E-05	5.24E-04	2.12E-04	4.63E-04	8.70E-05	2.08E-03	7.78E-04
**Co**	5.90E-05	6.17E-05	7.78E-06	2.75E-04	5.22E-04	4.79E-04	2.34E-03	1.69E-03	2.58E-03	1.50E-03

Figure S1: Results from the LM (low metal) lysis experiments. Metals are placed in order of decreasing concentrations measured for the methanogen, *M. jannaschii* [*Mj*]; concentrations for *P. furiosus* [*Pf*] are plotted relative to these. LM experiments were not conducted for *E. coli*. For each microorganism, values from all three lysis methods were averaged and used to calculate ± 1σ error bars, which are shown in the bottom graph of the figure panel. Lysis methods for all cells: U, ultrasonication; F, freeze-thaw; BB, bead beating.

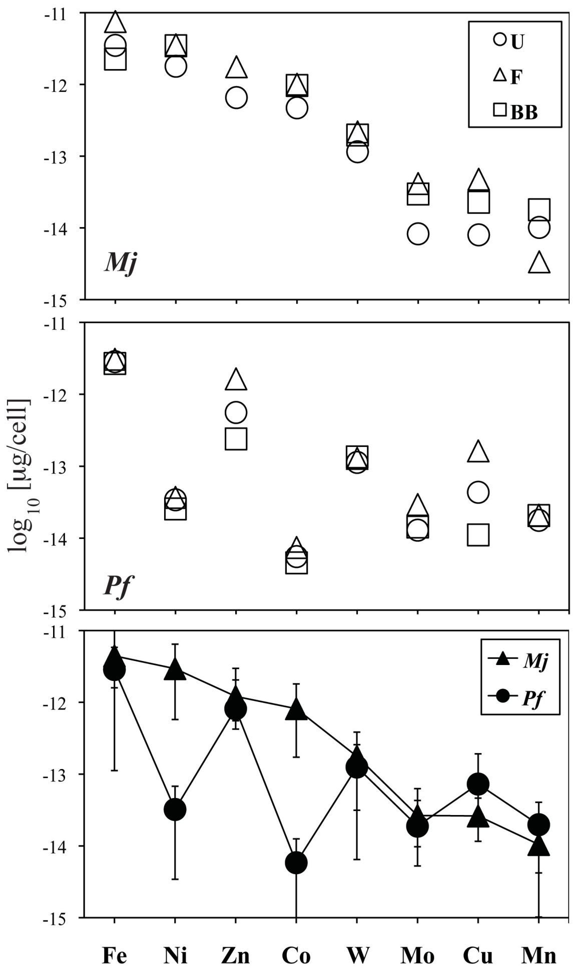

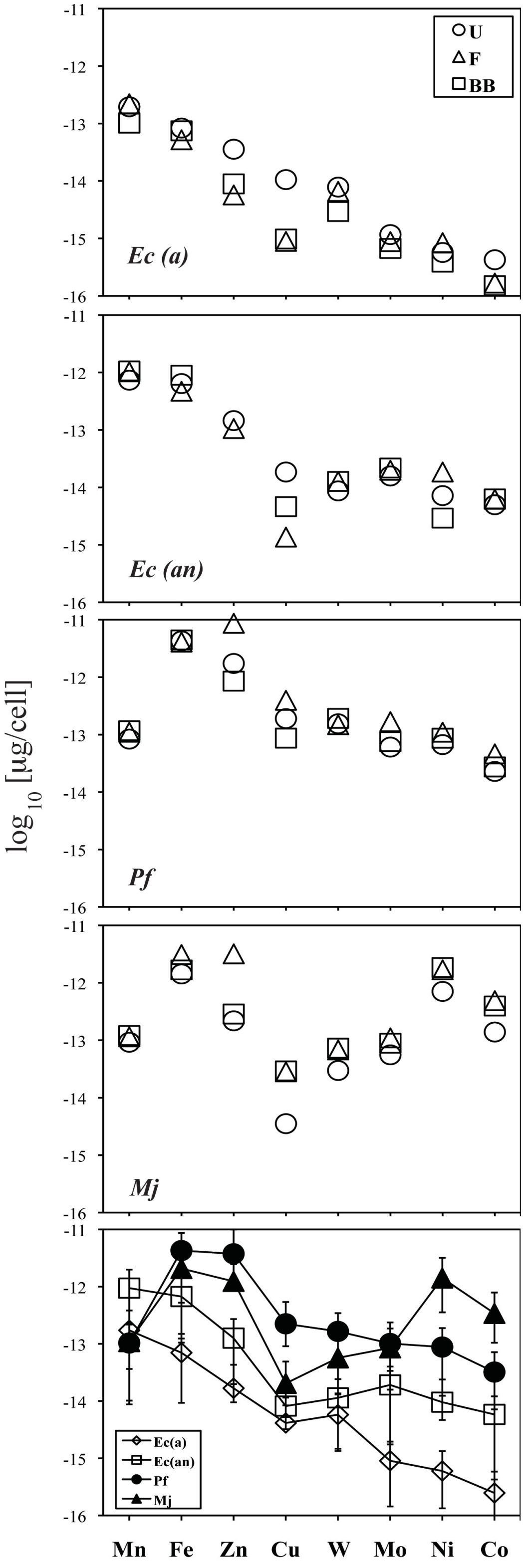

Figure S2: Results from the HM (high metal) lysis experiments. Metals for all graphs (bottom panel) are placed in order of decreasing concentrations measured for aerobic *E. coli (a)*. Metal concentrations for *E. coli* anaerobic [*Ec (an)*], *P. furiosus* [*Pf*] and *M. jannaschii* [*Mj*] are plotted relative to the order of the *E. coli (a)* values. For each microorganism, values from all three lysis methods were averaged and used to calculate ± 1σ error bars, which are shown in the bottom graph of the figure panel. Lysis methods: U, ultrasonication; F, freeze-thaw; BB, bead beating.

Table S3: Media volumes and cell density data for the LM and HM experiments.

Microorganism metal experiment	Media volume (ml)	Cell density (cells/ml)
Ec(a) - HM	1000	1.05×10^{11}
Ec(an) - HM	1000	7.62×10^{9}
Pf - LM	1000	7.93×10^{9}
Pf - HM	1000	5.68×10^{9}
Mj - LM	1000	6.16×10^{8}
Mj - HM	1000	2.91×10^{9}

Cell densities were determined via direct cell counting. Calculation of cell numbers for individual fractions involves the total medium volume and cell density reported above, as well as the number of fractions that the respective pellet was divided into for lysis and/or acid digestion (see text - Table 2). *E. coli* aerobic [*Ec* (a)]; *E. coli* anaerobic [*Ec* (an)]; *P. furiosus* [*Pf*]; *M. jannaschii* [*Mj*]. Experimental trace metal conditions: LM (low metal); HM (high metal).
