# Supplementary material for: A First Analysis of Metallome Biosignatures of Hyperthermophilic Archaea
Source: Archaea. 2012 Dec 3;2012:789278. doi: 10.1155/2012/789278 (PMC3518089; doi:10.1155/2012/789278)
Supplement: Supplementary file 2 [file 789278.f2.pdf]

Supplemental Data

Lysis data are reported below for the low metal (LM) and high metal (HM) experiments. The LM experiments involved only *P. furiosus* and *M. jannaschii* (Tables S1, S2, S3; Figure S1). In addition to these hyperthermophiles, the HM experiments also included *E. coli* grown aerobically and anaerobically (Tables S1, S2, S3; Figure S2). The three physical lysis methods utilized were ultrasonication, freeze-thaw and bead beating. Details of all methods are described in the manuscript text.

Compared to the trace metal concentrations measured in the growth media (Table S1), the wash steps undertaken prior to cell harvesting were generally effective in removing adsorbed metals (Table S2). Some small variations in metal concentrations are observed in the LM and HM experiments for the hyperthermophiles (Figures S1, S2) but the trends in the order of metal abundances are similar. Metal abundances for *P. furiosus* are in the order Fe > Zn > W, Cu, Ni > Mo, Mn, Co while the hierarchy of concentrations for the methanogen is Fe, Ni > Zn, Co > W, Mo > Cu, Mn. These patterns are also similar to those reported for the no metal (NM) experiments (Figures 1, 2). In the same vein, abundances of metals for *E. coli* (aerobic and anaerobic) are consistent in both NM and HM experiments. Zn and Cu displayed the most variation between lysis techniques under both LM and HM conditions across all microorganisms and may be attributable to factors such as storage and retention in membrane fractions, as alluded to in the text.

Of all the metals measured for *M. jannaschii*, Mn was present at the lowest concentration under LM conditions but increased an order of magnitude under HM concentrations. A similar increase was noted between the *E. coli* NM and HM experiments. Even though it is not commonly included when discussing the typical suite of important trace metals, Mn is required for a number of redox and nonredox processes as well as the metalloproteins (oxidoreductases, kinases, DNA and RNA polymerases, sugar transferases and peptidases) that facilitate these functions [1]. Studies in yeast demonstrate Mn uptake and intracellular trafficking are handled by cell surface and intracellular Mn-specific transporters and chaperones which are downregulated or upregulated in response to Mn surplus (toxicity) or deficiency [2]. However, Mn toxicity is of significant concern and may be a primary reason for such tight cellular control of this metal. One particular oxidoreductase, Mn superoxide dismutase (SOD), was shown to have important roles in *E. coli* for protection against heavy metal toxicity and oxygen stress [3]. The same study suggest that SODs may also have an additional role in sequestering and detoxifying Mn in bacteria in a similar manner as yeast (1, 2, 4). The cellular concentration of Mn in *E. coli* is maintained at a relatively low level of ~10 μM [5]. In the present study, the cells in the HM experiments were exposed to Mn concentrations in excess of 10 μM which could have induced some type of sequestration/detoxifying process, thus accounting for the elevated concentrations of Mn.

References

[1] V. C. Culotta, M. Yang, and M. D. Hall, "Manganese transport and trafficking: Lessons learned from *Saccharomyces cerevisiae*," *Eukaryotic Cell*, vol. 4, pp. 1159-1165, 2005.

[2] A. R. Reddi, L. T. Jensen, and V. C. Culotta, "Manganese homeostasis in *Saccharomyces cerevisiae*," *Chemical Reviews*, vol. 109, pp. 4722-4732, 2009.

[3] C. Geslin, J. Llanos, D. Prieur, and C. Jeanthon, "The manganese and iron superoxide dismutases protect Escherichia coli from heavy metal toxicity," *Research in Microbiology*, vol. 152, pp. 901-905, 2001.

[4] M. Yang, L. T. Jensen, A. J. Gardner, and V. C. Culotta, "Manganese toxicity and *Saccharomyces cerevisiae* Mam3p, a member of the ACDP (ancient conserved domain protein) family," *Biochemical Journal*, vol. 386, pp. 479-487, 2005.

[5] L. A. Finney, and T. V. O'Halloran, "Transition metal speciation in the cell: Insights from the chemistry of metal ion receptors," *Science*, vol. 300, pp. 931-936, 2003.

Figures and Tables

Table S1: Metal concentrations [mg/L] in growth media utilized for the low (LM) and relatively higher (HM) trace metal experiments. The LM lysis experiments were only carried out for the hyperthermophiles, *P. furiosus* and *M. jannaschii*. Concentrations were measured via ICP-MS. Values for Cu for *M. jannaschii* are below detection (BD) at a detection limit of 0.05 μg/L.

LM.

Metal	*P. furiosus*	*M. jannaschii*
**Mn**	6.33×10^{-1}	6.06×10^{-1}
**Fe**	4.03×10^{-1}	3.81×10^{-1}
**Zn**	3.95×10^{-1}	2.76×10^{-1}
**Mo**	4.35×10^{-2}	4.25×10^{-2}
**Ni**	7.13×10^{-2}	5.94×10^{-2}
**Cu**	5.14×10^{-2}	BD
**W**	7.66×10^{-2}	1.10×10^{-1}
**Co**	4.43×10^{-2}	4.17×10^{-2}

HM.

Metal	*E.coli* (a)	*E.coli* (an)	*P. furiosus*	*M. jannaschii*
**Mn**	2.23	1.90	2.04	1.93
**Fe**	3.06	2.61	1.30	1.14
**Zn**	8.01×10^{-1}	3.31×10^{-1}	1.22	1.12×10^{-1}
**Mo**	1.47×10^{-1}	1.73×10^{-1}	1.86×10^{-1}	1.61×10^{-1}
**Ni**	1.85×10^{-1}	1.63×10^{-1}	2.02×10^{-1}	1.87×10^{-1}
**Cu**	1.14×10^{-1}	1.22×10^{-3}	1.68×10^{-1}	BD
**W**	1.79×10^{-1}	2.06×10^{-1}	2.38×10^{-1}	2.05×10^{-1}
**Co**	1.66×10^{-1}	1.52×10^{-1}	1.50×10^{-1}	1.30×10^{-1}

Table S2: Metal concentrations [mg/L] in NaCl wash solutions after cells were washed. Concentrations were measured in samples of the wash solutions which were taken after cells were washed, centrifuged and pelleted (see text methods). For most experiments, data are for the solutions taken after wash step 2, but for **M. jannaschii* LM and HM, the results shown are from wash solutions taken after the third (final) wash. Concentrations were measured by ICP-MS. A number of values were below detection (BD), for which the following detection limits (μg/L) are as follows: Mn [0.01-0.5]; Fe [1.0-5.0]; Zn [0.1-5.0]; Ni [0.3-1.0]; Cu [0.01-0.05]; W [0.01].

Metal	NM				LM		HM			
	***E.coli* (a)**	***E.coli* (an)**	***P. furiosus***	***M. jannaschii***	***P. furiosus***	****M. jannaschii***	***E.coli* (a)**	***E.coli* (an)**	***P. furiosus***	****M. jannaschii***
**Mn**	3.70×10^{-4}	1.53×10^{-3}	BD	2.55×10^{-3}	4.65×10^{-3}	6.20×10^{-4}	4.02×10^{-1}	2.68×10^{-1}	2.83×10^{-2}	3.38×10^{-2}
**Fe**	3.05×10^{-3}	1.81×10^{-2}	BD	BD	BD	BD	2.80×10^{-2}	3.73×10^{-2}	BD	BD
**Zn**	BD	BD	BD	5.46×10^{-3}	6.07×10^{-2}	6.34×10^{-3}	1.13×10^{-2}	6.58×10^{-3}	1.42×10^{-1}	BD
**Mo**	1.06×10^{-3}	8.03×10^{-5}	5.23×10^{-5}	8.44×10^{-5}	9.05×10^{-4}	7.50×10^{-5}	7.55×10^{-3}	2.25×10^{-3}	3.60×10^{-3}	7.49×10^{-3}
**Ni**	1.72×10^{-4}	BD	BD	BD	BD	BD	2.91×10^{-3}	1.05×10^{-3}	3.97×10^{-4}	1.23×10^{-3}
**Cu**	5.92×10^{-4}	1.18×10^{-3}	BD	BD	5.34×10^{-3}	BD	2.66×10^{-3}	4.82×10^{-4}	1.36×10^{-2}	BD
**W**	BD	BD	1.70×10^{-5}	2.19×10^{-5}	5.24×10^{-4}	2.12×10^{-4}	4.63×10^{-4}	8.70×10^{-5}	2.08×10^{-3}	7.78×10^{-4}
**Co**	5.90×10^{-5}	6.17×10^{-5}	7.78×10^{-6}	2.75×10^{-4}	5.22×10^{-4}	4.79×10^{-4}	2.34×10^{-3}	1.69×10^{-3}	2.58×10^{-3}	1.50×10^{-3}

Figure S1: Results from the LM (low metal) lysis experiments. Metals are placed in order of decreasing concentrations measured for the methanogen, *M. jannaschii* [*Mj*]; concentrations for *P. furiosus* [*Pf*] are plotted relative to these. LM experiments were not conducted for *E. coli*. For each microorganism, values from all three lysis methods were averaged and used to calculate ± 1σ error bars, which are shown in the bottom graph of the figure panel. Lysis methods for all cells: U, ultrasonication; F, freeze-thaw; BB, bead beating.

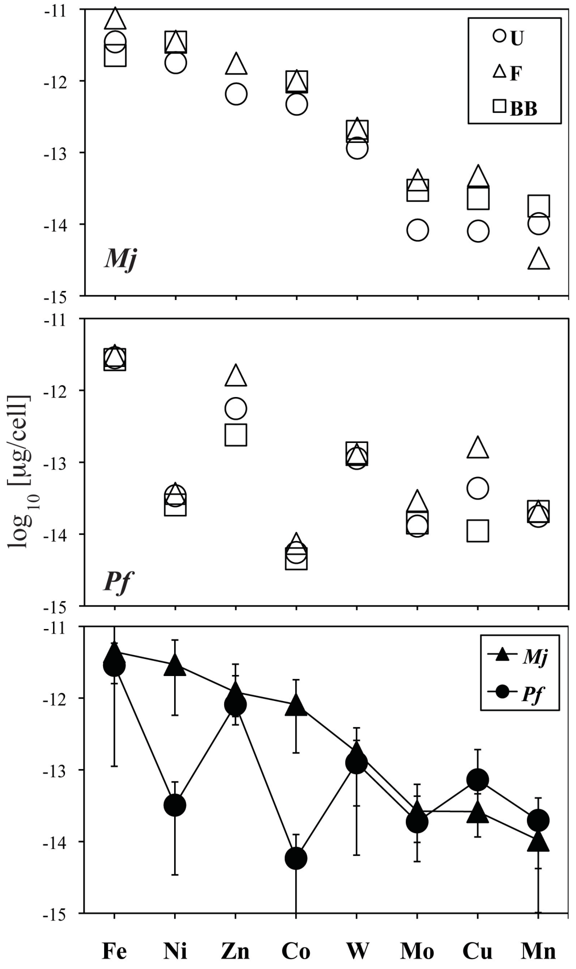

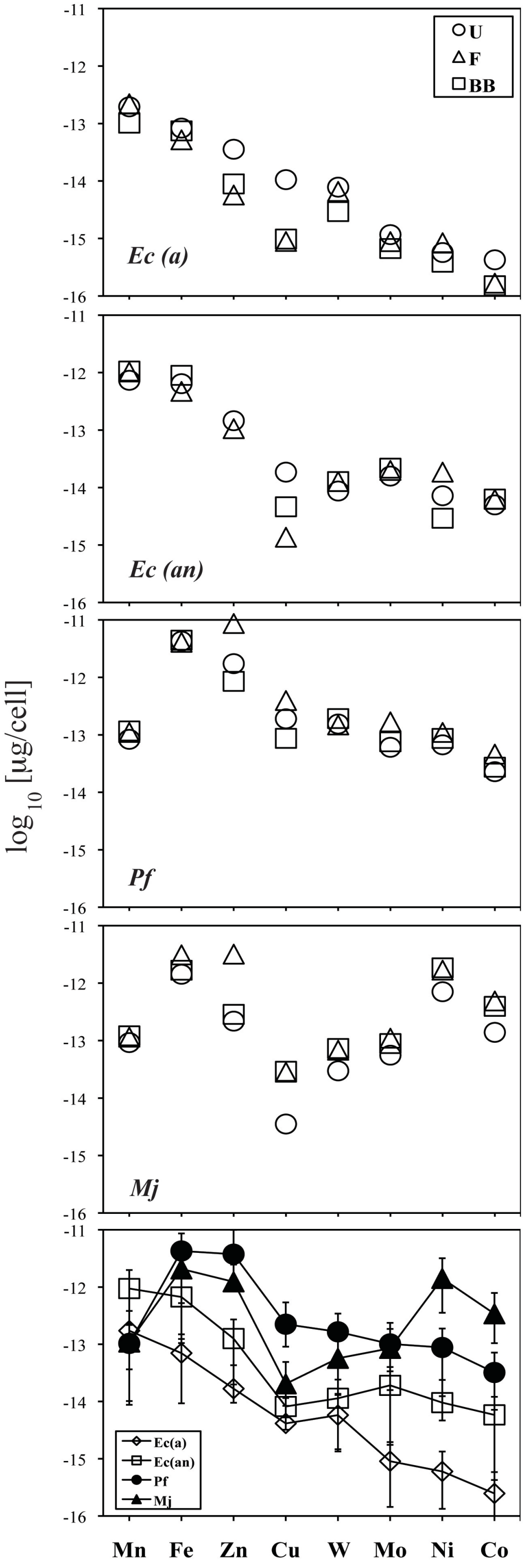

Figure S2: Results from the HM (high metal) lysis experiments. Metals for all graphs (bottom panel) are placed in order of decreasing concentrations measured for aerobic *E. coli (a)*. Metal concentrations for *E. coli* anaerobic [*Ec (an)*], *P. furiosus* [*Pf*] and *M. jannaschii* [*Mj*] are plotted relative to the order of the *E. coli (a)* values. For each microorganism, values from all three lysis methods were averaged and used to calculate ± 1σ error bars, which are shown in the bottom graph of the figure panel. Lysis methods: U, ultrasonication; F, freeze-thaw; BB, bead beating.

Table S3: Media volumes and cell density data for the LM and HM experiments.

Microorganism metal experiment	Media volume (ml)	Cell density (cells/ml)
Ec(a) - HM	1000	1.05×10^{11}
Ec(an) - HM	1000	7.62×10^{9}
Pf - LM	1000	7.93×10^{9}
Pf - HM	1000	5.68×10^{9}
Mj - LM	1000	6.16×10^{8}
Mj - HM	1000	2.91×10^{9}

Cell densities were determined via direct cell counting. Calculation of cell numbers for individual fractions involves the total medium volume and cell density reported above, as well as the number of fractions that the respective pellet was divided into for lysis and/or acid digestion (see text - Table 2). *E. coli* aerobic [*Ec* (a)]; *E. coli* anaerobic [*Ec* (an)]; *P. furiosus* [*Pf*]; *M. jannaschii* [*Mj*]. Experimental trace metal conditions: LM (low metal); HM (high metal).
